# Surgical treatment strategies for giant inguinoscrotal hernia – a case report with review of the literature

**DOI:** 10.1186/s12893-017-0331-x

**Published:** 2017-12-19

**Authors:** Julia Isabelle Staubitz, Peter Gassmann, Daniel Wilhelm Kauff, Hauke Lang

**Affiliations:** Department of General, Visceral and Transplantation Surgery, University Medical Center of the Johannes Gutenberg-University, Langenbeckstraße 1, 55131 Mainz, Germany

**Keywords:** Hernia, Inguinal hernia, Hernia repair, Components separation

## Abstract

**Background:**

An inguinoscrotal hernia is defined as “giant” if descending below the midpoint of the inner thigh of a patient in upright position. In developed countries this is a rare entity. In the literature different surgical techniques have been reported so far to achieve a successful treatment.

**Case presentation:**

We present the case of a 63 year-old man suffering from a giant inguinoscrotal hernia, whom we treated using a combined open transabdominal and inguinal approach following an unsuccessful laparoscopic attempt. Meshes were placed in a premuscular position (Lichtenstein’s procedure) and in a preperitoneal position. In addition, a reconstruction of the abdominal wall by modified components separation technique was performed. During the early postoperative period no complications were registered. Intensive care treatment was not necessary. The patient was discharged on postoperative day 8 in an excellent condition. Six months after surgery a scrotal hematocele was diagnosed and operatively removed. After a follow-up of 1.5 years neither hernia recurrence, nor chronic groin pain were recorded. The patient reported to be sexually active. His quality of life improved notably.

Additionally, a Medline and PubMed database research was performed to create an overall picture of the existing surgical treatment strategies. Included were patients with diagnosis of primary giant inguinoscrotal hernia according to the given definition. Emergency interventions and cases without details of the surgical approach were excluded.

**Conclusions:**

Firstly, this report describes a novel, successful surgical treatment of a giant inguinoscrotal hernia without administering preoperative progressive pneumoperitoneum therapy or visceral resection. Secondly, we summarize cases previously reported as a practical guide for possible surgical therapy approaches.

## Background

To be classified a giant inguinoscrotal hernia, the entity described has to extend below the level of the midpoint of the patient’s inner thigh in upright position [1] or should display an anteroposterior diameter of at least 30 cm or a laterolateral diameter of about 50 cm with non-reducibility for more than 10 years [2].

The prevalence of giant inguinoscrotal hernias in developed countries is very low and often associated with mental neglect for many years. If persisting for decades, a so-called *loss of domain* can occur, which illustrates that reintegration of the hernia’s content into the abdominal cavity is associated with severe problems related to the prevailing lack of space. The sudden elevation of the intraabdominal pressure can gravely impair the patient’s respiratory function. Moreover, an abdominal compartment syndrome can emerge, bringing along insufficient perfusion of the viscera [3].

In order to avoid the development of an abdominal compartment syndrome, the preoperative administration of a progressive pneumoperitoneum was suggested [4]. Studies show that this preoperative treatment can be useful, as the enlargement of the abdominal capacity can facilitate bowel reintegration as well as lung adaption to the postoperative situation in cases of a *loss of domain* [5, 6].

To avoid the development of a dangerously elevated intraabdominal pressure, also the reduction of the hernia’s content is possible. However, bowel resection is associated with the risk of anastomotic insufficiency. Another method to achieve tension-reduced abdominal occlusion, is the enlargement of the surface of the abdominal wall. For this purpose, components separation of the abdominal wall was already described in 1990 [7]. Also, direct midline laparotomy extension using mesh graft was successfully applied to surgically treat giant inguinoscrotal hernias [8].

## Case presentation

**Fig. 1:**
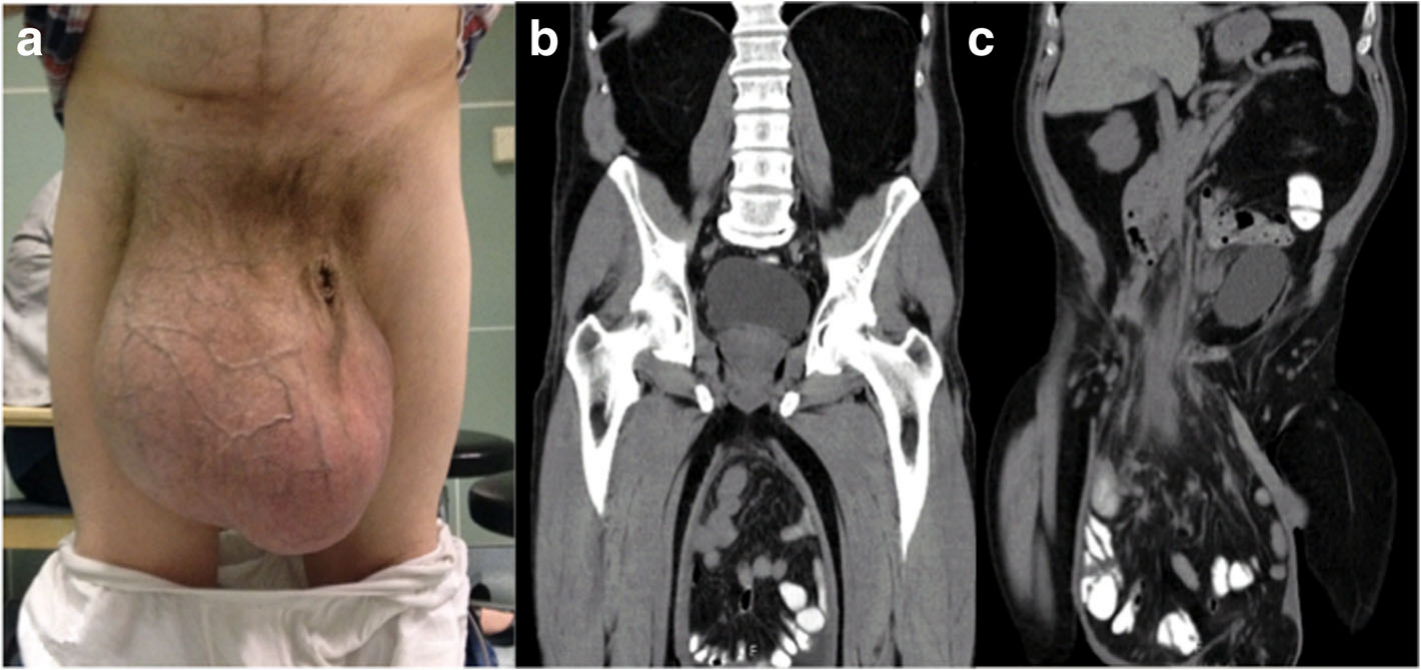
Frontal view of the giant inguinoscrotal hernia descending to the level of the knees (**a**). Computed tomography of the entity displaying subtotal evisceration of small bowel and ascending and transverse colon with intact vascularization (**b**/**c**)

A 63 year-old Caucasian patient who suffered from a giant inguinoscrotal hernia descending to his knees presented to our clinic. The entity had become more and more severe over the preceding decade. A computed tomography revealed that two-thirds of the small bowel and part of the ascending and transverse colon were included in the enormous herniation (Fig. 1). No incarceration was registered, nor did the patient suffer from groin pain or digestion irregularities. His waist circumference was 108.5 cm. No additional health problems other than arterial hypertension were registered. His laboratory parameters at presentation were normal (Hemoglobin: 0.009 mmol/L (normal range: 0.008-0.011 mmol/L); Leucocyte count: 7.5 × 10^9^/L (normal range: 3.5–10 × 10^9^/L), C-reactive protein: 19.05 nmol/L (normal range: <47,62 nmol/L)). An operation was suggested, since the giant inguinoscrotal hernia impaired his quality of life. The patient was married and was employed as freight worker when developing his medical condition. The entity notably limited his sexual activity. After a detailed explanation of possible risks including bowel resection and orchiectomy, the patient signed an informed consent for operative treatment. Although the possibility of preoperative administration of progressive pneumoperitoneum therapy was discussed, the patient refused this approach.

A laparoscopic reduction of the hernia content was unsuccessful. Therefore, an open transabdominal approach by midline laparotomy was chosen. The small intestine was replaced, but the right hemicolon remained fixed in the hernial sac. An additional right-sided inguinal incision to isolate the peritoneal sac was performed. By omental resection, we sufficiently gained space to mobilize the protruded colon back into the abdominal cavity. Complete resection of the hernial sac required the removal of the vascularization of the right testicle. Therefore, ipsilateral orchiectomy was performed. Subsequently, we used Lichtenstein’s technique in order to reinforce the abdominal occlusion using an UltraPro Mesh (10 × 15 cm, Ethicon, Johnson and Johnson, Norderstedt, Germany).

Via the midline laparotomy, we additionally placed a ProGrip Mesh (10 × 15 cm, Covidien, Dublin, Ireland) into a preperitoneal position, to thoroughly cover the instable area. Since the former content of the herniation was too voluminous to allow direct tension-free suturing of the lower part of the laparotomy wound, we subsequently performed modified components separation completed by mesh insertion. Therefore a subcutaneous tissue flap was released to the sides until anterior axillar line, followed by separation of the external oblique muscle from the internal oblique muscle and relaxing incisions of the internal oblique/transversus abdominis muscle. Bilateral access to the rectus sheath allowed the preparation of a retromuscular plane. After suturing of the posterior lamina (Vicryl), the reconstruction was completed by retromuscular mesh insertion (UltraPro Mesh 30 × 15 cm, Ethicon, Johnson and Johnson Norderstedt, Germany). The created overlap of the meshes (retromuscular and preperitoneal mesh) was 3 cm in the distal part. Suction drainages were placed subcutaneous plane. During the early postoperative period no complications occurred. Intensive care treatment was not necessary. No impairment of respiration or oxygenation was registered. We discharged the patient on postoperative day 8 in an excellent condition.

Six months after the operation, a hematocele in localization of the former right testicle was diagnosed. The operative removal of the hematoma and adjacent tissue was performed. After having removed the hematoma, the patient was fully satisfied with the overall postoperative result. Plastic surgery, in order to reduce the size of the scrotal skin surface, was not performed at any time. We successfully relied on the skin’s capacity to retract after removing the continuous tension caused by the giant hernia.

**Fig. 2:**
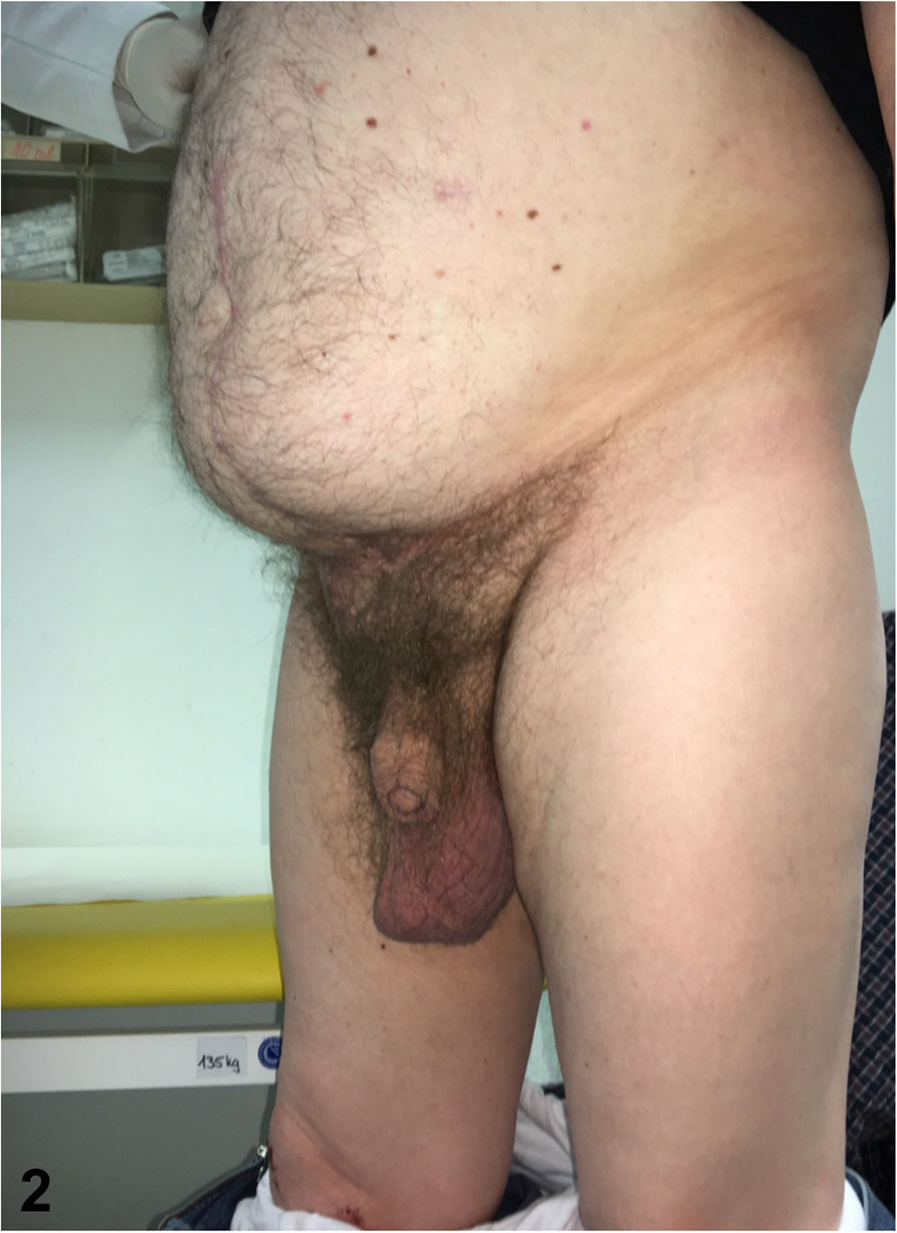
Clinical result following surgical treatment of giant inguinoscrotal hernia at 1.5 year postoperative follow-up

In a follow-up visit, 18 months after hernioplasty, no impairment of digestion was reported. Clinically and sonographically no hernia recurrence was registered. An acceptable cosmetic result prevailed (Fig. 2). Furthermore, the patient reported to be sexually active again. His quality of life had improved notably after the restoration of the giant inguinoscrotal hernia.

## Discussion and conclusion

Giant inguinoscrotal hernias represent a rare entity in developed countries. Different approaches are possible. Open abdominal and inguinal approaches are commonly used, if necessary in combination. According to the outer circumstances, ranging from high-end surgery in developed countries to surgery with limited resources in less developed countries, the surgical therapy has to be adapted to achieve the optimal result for the individual patient.

It is necessary to treat inguinoscrotal hernias, since organ perforation can occur, potentially causing peritonitis and sepsis [9, 10]. It is proved that early elective operations are associated with less fatal complications than emergency interventions [11]. Early elective surgical treatment helps to avoid visceral resection, which may bring along the risk of anastomotic leakage and potential prosthetic infection [12, 13].

In order to avoid the development of an abdominal compartment syndrome, resulting from a sudden elevation of the intraabdominal pressure following organ reposition, the preoperative administration of progressive pneumoperitoneum therapy was suggested [4]. Intraperitoneal gas insufflation can be performed continuously or fractionally. Atmospheric air should be preferred to O_2_ or CO_2_, since these gasses are absorbed rapidly [5]. Furthermore, one should consider that the application of this method requires a prolonged stay in hospital [14]. The insufflated gas can also spread into the hernial sac and, instead of widening the abdominal space, provoke an enlargement of the hernia itself [8]. After comprehensively informing our patient about this procedure and the associated risks, he refused this approach.

Another method to achieve tension-reduced abdominal occlusion is the enlargement of the abdominal space. This can be achieved by components separation of the abdominal wall, as reported by Ramirez et al. in 1990 [7]. For this technique, the rectus muscle is released from the posterior rectus sheath by separation of the external oblique muscle from the internal oblique muscle in an avascular plane, avoiding the need of mesh insertion. For reconstruction of the abdominal wall, in cases of giant inguinoscrotal hernias, also the direct extension of a midline laparotomy defect using mesh insertion was reported [8]. In the present case, we partly combined these techniques and additionally inserted a premuscular mesh in Lichtenstein’s position.

To avoid the development of a dangerously elevated intraabdominal pressure, reduction of the hernia’s content is another possible solution, e.g. by bowel resection. This, however, is associated with the risk of insufficiency of the anastomosis created, possibly leading to peritonitis, sepsis and even death. Anastomotic insufficiency can also cause infection of the mesh grafts inserted, potentially endangering the reconstruction of the abdominal cavity. In the case we presented, omental resection was performed, whereas bowel resection was avoided.

A laparoscopic approach, via transabdominal preperitoneal hernia repair (TAPP) or totally extraperitoneal (TEP) inguinal hernia repair, can be attempted to make use of the advantages of minimally invasive surgery [15]. If a laparoscopic approach is aimed for, it is recommendable to reduce the volume of the herniated organs prior to the operation, to facilitate the reposition manoeuvre. The application of Macrogol, (polyethylene glycol)-based laxatives, has been reported to be helpful in this regard, as it promotes the emptying of the intestine to be replaced [16].

Often, orchiectomy becomes necessary when removing the hernial sac because of adhesions, which are frequently observed in patients with a longer history of a giant inguinoscrotal hernia. Another reason for orchiectomy is the possible development of orchitis after an extended dissection of the spermatic cord. Additionally, orchiectomy was reported to facilitate an adequate closure of the hernial defect [14].

A follow-up analysis of cases of surgically treated giant inguinoscrotal hernias (from 1 to 96 months postoperatively) did not show recurrence, even though different approaches were performed (Table 1). The most infrequent approach is the singularly laparoscopic one (TAPP), reported by Momiyama et al. in 2016, who applied Stoppa’s method of placing a mesh in a pre-peritoneal position.

**Table 1:** Comparison of different surgical approaches to giant inguinoscrotal hernias

Bowel preparation	Pneumoperitoneum	Laparoscopic approach	Open abdominal approach	(Extended) inguinal approach	Omentectomy	Bowel resection	Orchiectomy	Scrotum resection	Components separation	Mesh in premuscular position	Mesh in preperitoneal postition	Mesh between internal and external oblique	Appendectomy	Postoperative intensive care	Postoperative discharge day	Hematocele/seroma development	Recurrence	Last follow-up (postoperative month)	
–	–	+	+	+	+	–	+	–	+	+	+	+	–	–	8	+	–	18	Present Case Report
–	+	–	–	+	–	–	–	–	–	+	–	–	–	n/a	n/a	–	n/a	n/a	Baca-Prieto et al. 2017 [19]
–	–	–	–	+	7×	–	1	–	–	+	–	–	2×	n/a	6.3*	10×	–	48	Bierca et al. 2013 [20] (15 cases)
+	–	–	+	+	–	+	+	+	–	–	+	–	–	+	9	–	–	18–96	Cavalli et al. 2015 [2]
+	–	–	+	+	–	+	+	+	–	–	+	–	–	+	8	–	–		Cavalli et al. 2015 [2]
+	–	–	–	+	–	–	+	+	–	–	+	–	–	+	7	–	–		Cavalli et al. 2015 [2]
+	–	–	–	+	–	–	+	+	–	–	+	–	–	+	6	–	–		Cavalli et al. 2015 [2] (2 cases)
–	–	–	–	+	+	–	–	–	–	+	–	–	–	–	4	+	–	3	Dinesh et al. 2014 [21]
+	–	–	+	+	–	+	+	–	–	–	–	–	–	–	28	–	–	8	Fadiran et al. 1992 [22]
–	–	–	–	+	–	–	–	–	–	+	–	–	–	n/a	n/a	–	–	12	Gillellamudi et al. 2010 [23]
–	–	–	+	–	–	–	–	–	–	–	+	–	–	–	5	–	n/a	n/a	Goonetilleke et al. 2010 [24]
+	–	–	+	–	–	–	–	–	+	–	–	+	–	–	13	+	–	2	Hamad et al. 2013 [25]
–	–	–	–	+	+	–	+	+	–	+	–	–	–	–	n/a	–	–	36	Karthikeyan et al. 2014 [26]
–	+	–	–	+	–	–	+	–	–	–	+	–	–	+	7	–	–	36	Kovachev et al. 2010 [14]
–	–	–	+	+	–	–	+	–	–	–	+	–	–	–	7	–	–	24	Kovachev et al. 2010 [14]
–	–	–	–	+	+	+	–	+	–	+	–	–	–	+	8	–	n/a	n/a	Kumar et al. 2016 [27]
–	–	–	–	+	+	–	–	–	–	–	–	–	–	+	6	–	–	n/a	Mohamad et al. 2017 [28]
+	–	+	–	–	–	–	–	–	–	–	+	–	–	–	12	–	–	12	Momiyama et al. 2016 [16]
–	–	–	+	+	+	+	–	–	–	–	+	–	–	+	n/a	–	–	6	Monestiroli et al. 2007 [12]
–	–	–	+	+	+	+	+	+	–	–	+	–	–	+	n/a	–	–	12	Patsas et al. 2010 [29]
–	+	–	+	–	–	–	–	–	–	–	+	–	+	–	14	–	–	11	Piskin et al. 2010 [30]
–	+	–	–	+	–	–	–	–	–	–	+	–	+	–	13	–	–	9	Piskin et al. 2010 [30]
–	–	–	+	+	–	–	+	–	–	+	–	–	–	+	10	–	–	6	Sahsamanis et al. 2016 [31]
–	–	–	–	+	–	–	–	–	–	–	–	–	–	–	1	1×	–	1	Savoie et al. 2014 [18] {25 cases}
–	–	–	+	+	+	–	+	–	–	+	–	–	–	n/a	n/a	–	–	1	Singh et al. 2015 [32]
+	–	–	–	+	–	–	+	2×	–	–	4×	–	–	–	3	–	–	12	Sturniolo et al. 1999 [33] (5 cases)
–	–	–	–	+	+	–	–	–	–	+	–	–	–	–	7	+	–	48	Trakarnsagna et al. 2014 [34]
–	–	–	–	+	–	–	–	+	–	+	–	–	+	+	6	–	–	3	Turner et al. 2010 [35]
–	–	–	–	+	+	–	–	–	–	+	–	–	–	+	6	–	–	6	Tarchouli et al. 2015 [36]

n/a = not assessed, * = “on average” in cited source

In the literature, reduction of the scrotal skin is described as helpful to achieve a cosmetically acceptable result [2]. We successfully relied on the skin’s ability to recover after the removal of continuous tension caused by the giant hernia. Scrotal skin was not resected at any time. On the other hand, it was described as useful to preserve the redundant scrotal skin, in order to keep a safety net, which can allow the temporary replacement of hernial contents back into the scrotum. This may be necessary in the early postoperative period, after a failed hernia repair, or in case of respiratory compromise [17]. However, scrotal skin reduction seems to be protective with regard to the postoperative development of hematocele/seroma (Table 1). According to Savoie et al., who contributed a case series to the current literature, in which 25 men from the Ivory Coast underwent Bassini’s hernia repair, the abandonment of the distal hernial sac in the scrotum can be proposed to selected patients, in order to prevent bleeding and cutaneous complications [18].

In the present case, we successfully used a novel combination of the existing techniques avoiding bowel resection and preoperative progressive pneumoperitoneum.

## Abbreviations

TAPP: Transabdominal preperitoneal hernia repair
TEP: Totally extraperitoneal hernia repair
